# Unmasking biomarkers in small cell lung cancer: implication for precision oncology

**DOI:** 10.3389/fonc.2026.1842511

**Published:** 2026-06-29

**Authors:** Devshree Parganiha, Mohit Joshi, Sivaramasundaram Sankarasubramanian, Sharanya V Pandit, Harishmitha Nagaraj, Naveenkumar Perumal

**Affiliations:** School of Bio Sciences and Technology, Vellore Institute of Technology, Vellore, Tamil Nadu, India

**Keywords:** biomarkers, cancer stemness, miRNA, molecular heterogeneity, precision oncology

## Abstract

Small cell lung cancer (SCLC) ranks among the most aggressive cancers, posing significant challenges in early diagnosis and treatment with limited therapeutic options. Smoking is one of the major risk factors, which constitutes a substantial proportion of lung cancer cases globally. Despite extensive studies, little progress has been made in treating SCLC, and therefore a striking need for novel therapeutic strategies. Increased genetic and transcriptional heterogeneity has significantly complicated SCLC, including mutations in key regulators such as TP53, RB1, and MYC family genes. Genetic and Transcriptional heterogeneity of SCLC is further exacerbated by the expression of key regulators, including ASCL1, NEUROD1, YAP1, POU2F3, and ATOH1. Because of their unique biological properties, each distinct subtype exhibits a potential specific therapeutic targeting. Biomarkers act as therapeutic guidance in SCLC as they play an important role in early detection, diagnosis, and prognosis. Distinct functional biomarkers, such as genetic, circulating, biochemical, and imaging, provide valuable insights into tumor biology and patient management. Cancer stem cells (CSCs) are an emerging critical challenge in SCLC as they contribute to disease resistance and render the conventional therapies ineffective, leading to tumor recurrence, e.g., CD133, SOX2, and CD44, etc. This in-depth review provides insight into the current understanding of SCLC biology, encompassing its epidemiology, risk factors, genetic features, and molecular subtypes. It also focuses on recent therapeutic insights underlying the promising biomarkers and inhibitors targeting CSCs to overcome drug resistance and improve overall treatment outcomes in SCLC patients.

## Highlights

- Small-cell lung cancer remains a highly progressive neoplasm with adverse survival outcomes and scarce treatment options.
- Extensive genetic and molecular heterogeneity, including mutations in RB1, TP53 and MYC oncogene, mediates aggressive cancer growth and resistance to the existing treatment options.
- Genetic profiling revealed key regulators such as ASCL1, NEUROD1, YAP1, POU2F3 AND ATOH1, enabling precision therapeutics.
- Potential Biomarker including molecular, circulating, miRNA, LncRNA, aids in improving early detection of SCLC and provides personalized treatment options.
- Combining molecular diagnosis with the advanced targeted therapies may enhance prognosis in SCLC patients.

## 1 Introduction

**Figure 1:**
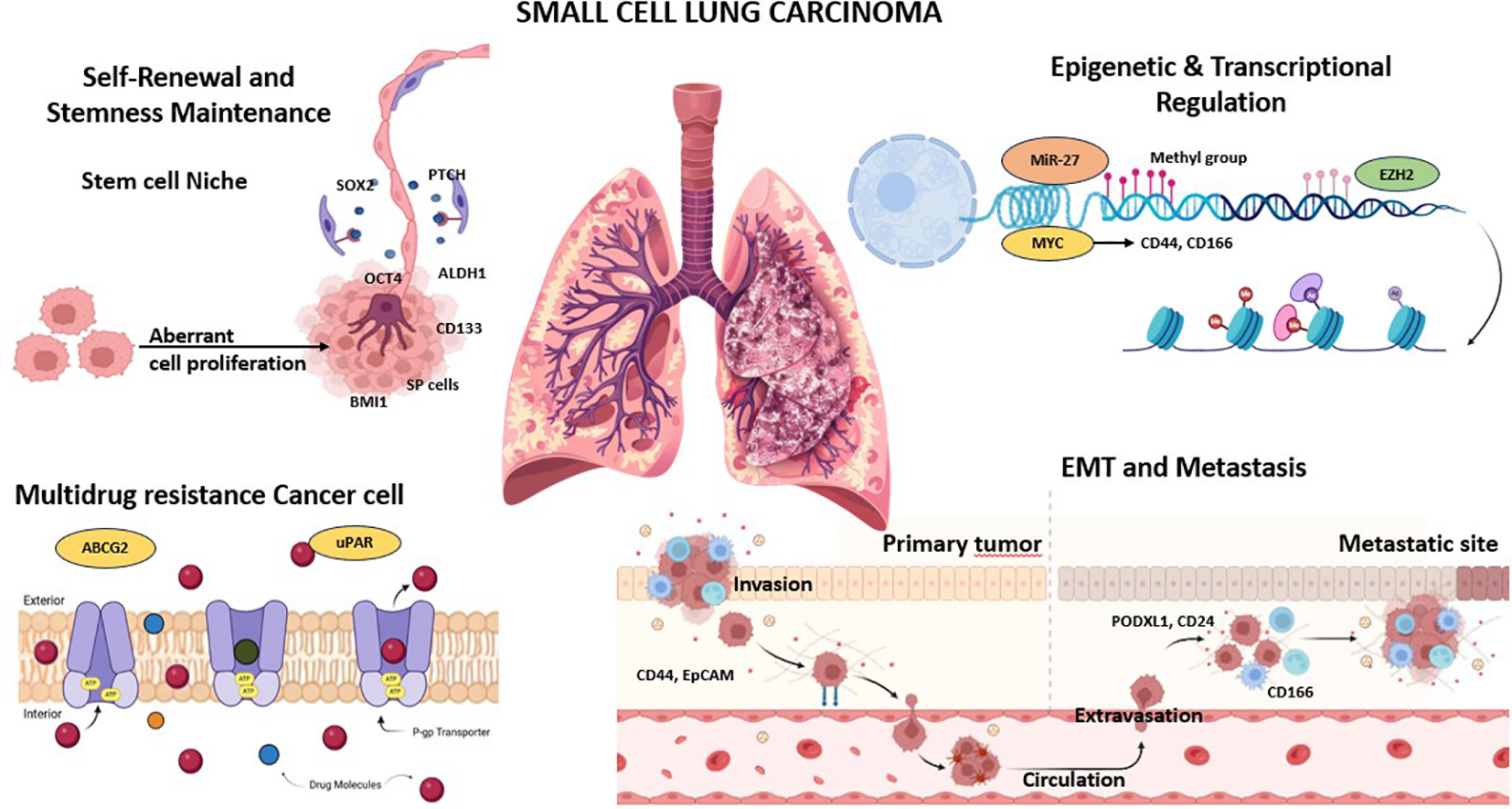
Representation of molecular pathways that are involved in SCLC progression, stemness maintenance, multidrug resistance, epigenetic & transcriptional regulation.

Neoplastic disease of the lung is the most commonly diagnosed cancer in the world, with 2,480,301 cases in 2022, accounting for about 12.4% of all cancers, and 1,817,172 deaths have been reported, making it one of the foremost drivers of cancer mortality based on the GLOBOCAN 2022 (1). Lung cancer in India accounts for an annual incidence of 72,510 cases (5.8%) and 66,279 deaths (7.8%) based on the GLOBOCAN 2022 (1). Based on microscopic appearance, lung cancers are histologically classified as non-small cell lung cancer (NSCLC) and small cell lung cancer (SCLC). SCLC is classified as a lung neuroendocrine neoplasm (NEN) and accounts for 15% of lung cancer, whereas NSCLC is classified as adenocarcinoma, squamous cell carcinoma, and large cell carcinoma, collectively accounting for approximately 85% of all lung cancer cases (2). SCLC remains a highly aggressive disease with limited survival outcomes, with improvements observed in limited-stage SCLC (LS-SCLC), where the 2-year relative survival increased from 36% in 2001–2002 to 46% in 2015–2016. However, outcomes for extensive-stage SCLC (ES-SCLC) remain poor, with 2-year survival rates below 10%. Median survival rarely exceeds one year, and the overall five-year survival rate remains under 7% (3). In most cases SCLC is diagnosed at a late stage, where it has metastasized to multiple organs such as the liver, brain, bone marrow, and adrenal gland, with a survival rate of 2 to 4 months (2). While these metastatic sites may exhibit common traits, the underlying disease mechanism, as well as the associated tumor microenvironment (TME), tend to exhibit considerable variation in these tumors. **Figure 1** represents the overall molecular landscape involved in SCLC heterogeneity. *De Novo* SCLC is more common among younger people and non-smokers, whereas transformed SCLC occurs when an NSCLC tumor phenotypically shift towards SCLC, often after treatment with targeted therapy or immunotherapy. NSCLC can also be transformed into SCLC, called “Transformed-SCLC (T-SCLC)” under targeted therapy, immunotherapy, and chemotherapy. Patients with loss of RB1 (Tumor suppressor gene) and development of resistance to EGFR-TKIs (drugs used in treating NSCLC) showed formation of T-SCLC (4). In about 95% of SCLC cases, tobacco smoking represents the leading risk factor in the development of lung cancer. There are also some environmental risk factors, includes chemicals such as chloromethyl esters that are used in manufacturing chemicals, asbestos, and radon. Occupation is also one of the factors; people working in occupations like coke oven workers, miners, road and railroad workers are often exposed to carcinogens, including chromium, silica dust, nickel, and arsenic that contribute to a higher risk of lung cancer (5).

Management of SCLC depends on its stage, with two categories: limited-stage (LS-SCLC) and extensive-stage (ES-SCLC), reflecting the tumor’s anatomical distribution and aggressive behavior (2). LS-SCLC is in the Ipsilateral hemithorax, is small, localized and often treated with radiation therapy (6). For LS-SCLC, initial therapy typically includes a therapeutic platinum compound (cisplatin or carboplatin) alongside etoposide and thoracic radiation (7). COCIS meta-analysis was done to identify the treatment efficacy of cisplatin versus carboplatin-based chemotherapy, which suggested no difference between the two agents for both ES and LS patients. However, severe neurological and renal toxicities as well as nausea and vomiting, were seen with cisplatin treatment, and increased frequency of high-grade hematologic adverse effects was associated with Carboplatin treatment (8). The combination of chemotherapy plus immunotherapy is the preferred option for treating ES-SCLC, as recommended by the National Comprehensive Cancer Network (NCCN) (9). Proton-Beam Therapy has also been explored in treating LS-SCLC patients, which showed a median overall survival (OS) of 28.2 months with fewer side effects (10). Lurbinectedin, an FDA-approved drug is used as a second-line treatment in patients with SCLC inhibiting gene transcription and RNA Polymerase II in SCLC patients (11). Delta-like protein 3 (DLL3), a negative regulator of the NOTCH signaling pathway, is highly expressed in the majority of SCLC patients. Rovalpituzumab tesirine (Rova-T), an anti-DLL3 antibody-drug conjugate, has demonstrated potent anti-tumor activity in patients with recurrent SCLC (12, 13). In 2018, patients with ES-SCLC demonstrated improved overall survival (OS) and progression-free survival (PFS) when treated with a combination of atezolizumab and chemotherapy (9). Based on the IMpower133 clinical trial, the FDA approved the use of atezolizumab in combination with carboplatin and etoposide for ES-SCLC in 2019 (14). Another strategy that has shown a significant OS benefit in ES-SCLC involves combining durvalumab with first-line chemotherapy (15). Immune checkpoint inhibitors, particularly those inhibiting PDL1/PD-1 like Pembrolizumab, Atezolizumab, and Durvalumab have been in trials showing significant improvement in the overall survival either alone or in combination with chemotherapy. Other small molecule inhibitors targeting topoisomerases, PARP, HDAC, Chk1 and Aurora kinase have been explored in other studies (3, 16). Despite these advances, therapeutic outcomes in SCLC remain limited, underscoring the need for novel strategies. The present work addresses the gaps through the identification and validation of new molecular targets and pathways related to therapeutic advancements in SCLC. The limited survival rate was seen with VEGF inhibitors and DDR-specific agents; there is a critical need to enhance therapeutic specificity and overcome resistance. Through investigating pathways involved in tumor progression, immune invasion and epigenetic regulation, this work may help to refine patient-specific (biomarker-driven approaches like SLFN11) response rates (13).

## 2 Genetics and common mutations of SCLC

SCLC cells exhibit an amalgamation of genetic abnormalities, including mutations, amplifications, insertions, deletions, and translocations. Genes commonly mutated in SCLC includes TP53, RB1, EGFR, CSMD3, and ADAMTS19. Furthermore, the existence of certain genetic signatures in SCLC, such as APOBEC-associated mutation patterns and DNA Damage Repair (DDR)-related signatures, has been revealed (17, 18). A study recently identified hub genes such as ITGA10, DUSP12, PTGS2, FOS, TGFBR2, and ICAM1 which further shed light on the molecular pathways underpinning SCLC formation (19). Males and females with SCLC have different gene expression signatures and mutation frequencies. Females are most likely to have RB1 mutations, whereas men have greater levels of AMPH, GRIN2C, and WNT4 expression. These genetic variations may contribute to documented sex differences in SCLC, with females having smaller tumors, fewer metastases, and lower stages, as well as higher overall survival compared to males (20). These observations contribute to a clearer understanding of SCLC genetics and may facilitate the advancement of precision therapies and tailored treatment options.

Genome-wide association analyses (GWAS) have revealed that specific genetic loci, particularly 15q25, 5p15, and 6p21, are linked to an increased susceptibility to lung cancer (21–23). Five single-nucleotide polymorphisms linked to increased risk of SCLC, although showing moderate or weak strength, were identified through meta-analysis: CHRNA5 rs16969968 (weak), CYP1A1 rs4646903 (moderate), GSTM1 present/null (moderate), NQO1 rs1800566 (weak), and XPC rs2228001 (weak) (24). According to a study reported by Tlemsani et al. (2021), (25), whole-exome sequencing of SCLC patients revealed inherited pathogenic variants in the genes RAD51D, CHEK1, BRCA2, and MUTYH (25). A region located on chromosome 15 (called 15q24-25.1) has been seen to be strongly linked to higher SCLC risk because these regions have receptor genes (CHRNA5, CHRNA3, CHRNB4) for tobacco chemicals (21, 26). Two specific SNPs (rs16969968 and rs578776) in this chromosomal area are also linked to the amount of cotinine (a nicotine metabolite) in a smoker’s body (27).

SCLC is known for extremely high mutation rates and genomic instability, likely associated with exposure to tobacco carcinogens (28). The molecular landscape of SCLC is marked by a variety of genetic modifications, including alterations in receptor tyrosine kinases and their downstream effectors, copy number variations (CNVs), and somatic transcription factor mutations, variations in tumor suppressor genes, and chromatin remodeling. TP53 and RB1 are the most frequently mutated genes in SCLC, with about 85% and 57% mutation frequencies, respectively (29). Next-generation sequencing has helped in identifying genetic alterations in SCLC, for example, 3p deletion, one of the most frequent chromosomal abnormalities (30). In conjunction with the amplification of MYC oncogenes (31), frequent inactivation of the Notch signaling pathway is also observed at high incidence (32). Some of the genes that are mutated in SCLC are summarized in the **Table 1**.

**Table 1:** Common genetic mutations in SCLC.

S. no	Gene	Type of defect	Biological impact	Reference
1	TP53	Inactivating mutation	Prevents cell cycle arrest and apoptosis; early event in SCLC	(33)
2	RB1	Inactivating mutation	Promotes uncontrolled cell cycle entry; early SCLC event	(33)
3	MYC	Amplification	Enhances cell proliferation	(34)
4	ASCL1	Overexpression	Promotes neuroendocrine differentiation	(34)
5	LRP1B	Inactivating mutation, loss of expression	Tumor suppressor; nuclear LRP1B may enhance invasion via NEAT1(Nuclear Enriched Abundant Transcript 1); potential immunotherapy biomarker	(35, 36)
6	MAP3K13	Overexpression	Oncogenic kinase; stabilizes mutant p53; regulates MYC via MAP3K13–TRIM25–FBXW7α axis	(35)
7	MSH6	Mutation	Impaired DNA mismatch repair mechanism; associated with better prognosis	(35, 37, 38)
8	SPEN	Inactivating mutation or altered expression	Act as a transcriptional repressor; linked to poor prognosis	(35, 39)
9	PIK3CA	Activating mutation	Activates PI3K-AKT pathway; promotes proliferation, metastasis and poor prognosis	(40–41)
10	FAT1	Mutation	Alters EMT and WNT signaling; promotes tumor progression and liver metastasis in SCLC	(35, 42)
11	KMT2	Inactivating mutation	Loss of enhancer activity; promotes tumorigenesis	(43)
12	CREBBP	Inactivating mutation	Alters chromatin remodeling; enhances plasticity	(43, 44)
13	PTEN	Deletion or loss of expression	Hyperactivation of PI3K/AKT/mTOR pathway; genomic instability	(43)
14	APC	Inactivating mutation or deletion	Leads to unregulated WNT signaling; increased proliferation and migration.	(40, 45, 46)
15	EGFR	Activating mutation	Constitutive activation of RAS/MAPK and PI3K/AKT pathways	(42)
16	ARID1A	Inactivating mutation	Loss of chromatin regulation; impaired DNA repair mechanisms	(47)
17	PTPRD	Inactivating mutation or deletion	Loss of negative regulation; enhanced proliferation	(48)
18	ATRX	Inactivating mutation or deletion	Telomere dysfunction; chromatin remodeling defects	(45)

## 3 Cellular origin of SCLC

Small-cell lung cancer (SCLC) is an aggressive neuroendocrine malignancy, characterized by inactivation of the tumor suppressors TP53 and RB1 (12). Emerging evidence suggests that a combination of genetic alterations and the cell-of-origin drives SCLC subtypes. Several studies have been carried out to precisely define the cellular origin of SCLC using *Trp53/Rb1* mouse models (49). Studies on cell type-restricted Adenoviral vectors expressing Cre gene under the promoter of CGRP, SPC & CC10 gene, with *Trp53^F/F^; Rb1^F/F^* mice demonstrated that Neuroendocrine (NE) cells are the predominant cell of origin of SCLC, mainly driven by CGRP promoter. Also, targeting Alveolar type II (AT2) cells with SPC promoter-driven Cre induces SCLC. It was also highlighted that targeting club secretory cells with CC10 promoter-driven Cre was resistant to transformation to SCLC. Also, Park et al., 2011 (50) reported CC10 and SPC promoter-driven Cre did not generate SCLC when deleting *Trp53* and *Rb1*, unlike Sutherland et al., 2011 (49). Using a lineage-tracing mouse model (CGRP-CreER crossed with ROSA26-mTmG and Trp53/Rb1 conditional strains) confirmed that lung NE cells develop SCLC (51). More recent studies using lineage-specific oncogene activation and chromatin-based cell-of-origin (COO) prediction indicate that basal cells and other epithelial lineages can be reprogrammed toward an SCLC fate upon acquisition of canonical SCLC driver mutations (52–53). This highlights the importance of both cell-of-origin and genetic context in shaping tumor phenotype and metastatic behavior. Perhaps recent research highlights that current SCLC therapeutics often develop resistance and tumor relapse, accentuating the need for a better understanding of genetic drivers and potential cells of tumor origin to develop more effective and durable treatment strategies (3).

## 4 Transition of NSCLC to SCLC

Transformation of NSCLC to SCLC is a distinct resistance mechanism towards targeted therapies, most particularly observed in EGFR-mutant lung adenocarcinomas when treated with TKIs. Non-mutant EGFR NSCLCs have also been shown to change lineage during immunotherapeutic interventions, particularly PD-1/PDL-1 inhibitors (54). This transition is driven by lineage plasticity rather than genetic changes but via transcriptional and epigenetic reprogramming involving EMT, stem-like features and histological transformation, acquiring resistance to EGFR TKIs. The newly transformed cells retain the original mutation of EGFR but loses the dependency on it, gaining neuroendocrine markers like synaptophysin, chromogranin A, and CD56. The transformed SCLCs are clinically more aggressive compared to the *de novo* SCLCs. Molecularly, transformation is commonly associated with concurrent loss of *TP53* and *RB1*, which facilitates neuroendocrine differentiation and phenotypic switching. Epigenetic reprogramming and transcriptional alterations involving PRC2 activation, upregulation of NOTCH and PI3K/AKT pathways, including upregulation of neuroendocrine markers (e.g., ASCL1 and NEUROD1), further support this transition (55). Importantly, transformed tumors often retain the original driver mutation but become insensitive to targeted inhibition, instead adopting therapeutic vulnerabilities, a characteristics of SCLC (56). Transformed SCLCs respond to platinum-etoposide (EP) treatments with an increase in the PFS rate of ~3 months, EP with EGFR TKI’s tend to show an increase in the PFS to ~5 months and EP with immunotherapy shows no increase in patient survival rates (57, 58). NSCLC transformation into SCLC reflects a lineage-driven escape from targeted therapy, marked by TP53/RB1 loss and neuroendocrine reprogramming. These aggressive tumors retain original driver mutations but shed their dependency, leaving platinum-based regimens as the mainstay with only modest benefit. This highlights an urgent need for biomarker-guided and novel therapeutic strategies to overcome this distinct resistance mechanism.

## 5 SCLC subtypes

**Figure 2:**
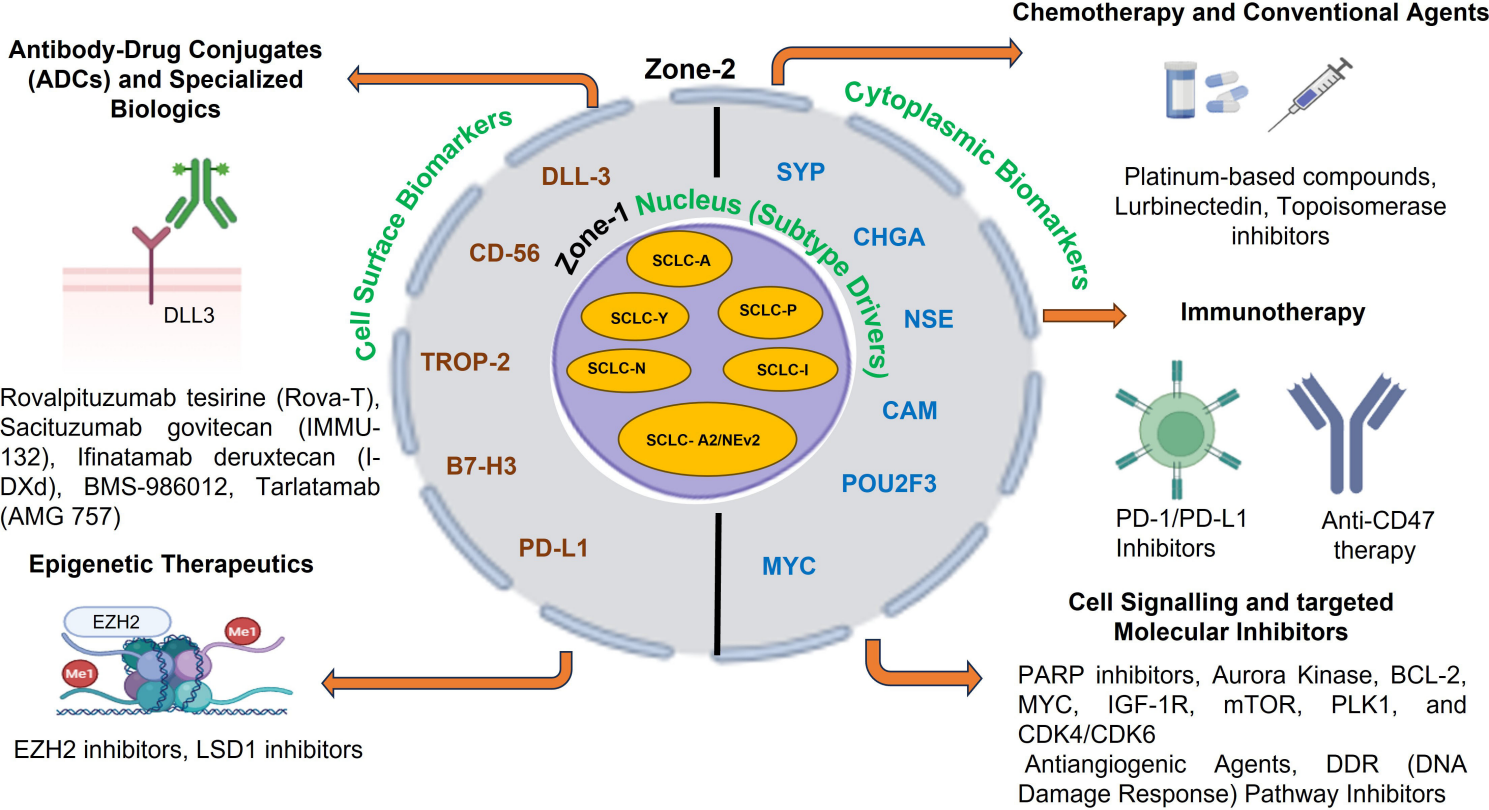
The integrated molecular and therapeutic landscape of small cell lung cancer (SCLC). The diagram provides a comprehensive overview of the two functional zones defining SCLC biology and its clinical management. Zone 1: Nucleus (Subtype Drivers): SCLC is categorized into molecular subtypes based on the expression of key transcription factors. These include SCLC-A (ASCL1: Achaete-scute homologue 1), SCLC-N (NEUROD1: Neuronal Differentiation 1), SCLC-P (POU2F3: POU Class 2 Homeobox 3), SCLC-Y (YAP1: Yes1 Associated Transcriptional Regulator), SCLC-I (Inflamed, marked by immune gene signatures), and the SCLC-A2/NEv2 neuroendocrine variant. Zone 2: Intracellular and Surface Biomarkers: Cell Surface Biomarkers: Key targets for therapeutic delivery and diagnosis include DLL3, Delta-like protein 3; CD-56 (NCAM), TROP-2, Trophoblast cell surface antigen 2;, B7-H3, and PD-L1, Programmed death-ligand 1; Cytoplasmic Biomarkers: Classic diagnostic neuroendocrine markers include SYP, Synaptophysin; CHGA, Chromogranin A; NSE, Neuron-specific enolase; CAM, CAM 5.2 cytokeratin; POU2F3, and MYC family amplifications. Therapeutic Strikes (External Inhibitors): Targeted Therapies - Chemotherapy: Standard regimens includes Platinum-based compounds (Cisplatin/Carboplatin), Lurbinectedin, and Topoisomerase inhibitors; Immunotherapy: Includes PD-1/PD-L1 Inhibitors (e.g., Atezolizumab, Durvalumab) and Anti-CD47 therapy; Targeted & Molecular Inhibitors: Comprised of PARP inhibitors (Talazoparib, Olaparib), Aurora Kinase inhibitors (Alisertib), Antiangiogenic Agents (Bevacizumab), and DDR, DNA Damage Response; Pathway Inhibitors; Epigenetic Therapeutics: Targeting chromatin remodeling via EZH2 inhibitors and LSD1 inhibitors; ADCs, Antibody-Drug Conjugates & Specialized Biologics: Includes Rova-T, Sacituzumab govitecan (IMMU-132), I-DXd, Ifinatamab deruxtecan, BMS-986012, and BiTE, Bispecific T-cell engager Tarlatamab (AMG 757). Other Cell Signaling Inhibitors: Potential targets include BCL-2, MYC, IGF-1R, mTOR, PLK1, and CDK4/CDK6.

According to histological classifications by the WHO and the NCCN, SCLC is categorized into two subtypes: pure small cell carcinoma (also known as oat cell carcinoma) and combined SCLC (2). Based on neuroendocrine features, SCLC can further be subdivided into neuroendocrine (NE) and non-neuroendocrine (non-NE) subtypes, as determined by immunohistochemical (IHC) staining for markers such as synaptophysin (SYP) and chromogranin A (CHGA) (59). Transcriptomic profiling of SCLC cell lines has facilitated the identification of additional subtypes based on transcription factor expression, a classification that has been corroborated by analyses of tumor samples (60–64). At the molecular level, considering transcription factors and immune-related gene expression, SCLC can be classified into six subtypes: SCLC-A, SCLC-A2, SCLC-N, SCLC-P, SCLC-Y, and SCLC-I (61, 65). **Tables 2** and **3** shows the subtypes of SCLC and inhibitors targeting them (**Figure 2**).

**Table 2:** Molecular heterogeneity of SCLC.

S. no.	Subtype	Key marker(s)	Key features	Reference
1	SCLC-A	ASCL1, CHGA, SYP, INSM1, NKX2-1 (TTF1)	NE subtype; regulates NE differentiation; MYCL expression; sensitive to therapy	(63, 66, 67)
2	SCLC-N	NEUROD1, CHGA, SYP, INSM1	NE subtype; promotes NE differentiation and cancer progression; MYC expression	(63, 67, 70)
3	SCLC-P	POU2F3	Non-NE subtype; tuft cell-like features	(73)
4	SCLC-Y	YAP1, TAZ, TEAD2/3, AJUBA	Non-NE subtype; Hippo pathway activation; high MYC expression; rare	(60, 73–75)
5	SCLC-A2/NEv2	ASCL1 + HES1	NE variant; associated with liver metastasis	(61, 62, 75–77)
6	SCLC-I	Immune-related genes (e.g., PD-L1, PDCD1, CTLA4, CCL5, CXCL10, HLA-DRB1)	Inflamed subtype; high immune infiltration; better response to immunotherapy	(61)

**Table 3:** Inhibitors targeting molecular heterogeneity of SCLC.

S. no.	Targets	Inhibitors	Reference
1	SCLC-A	BCL-2 inhibitor (Venetoclax) and LSD-1 inhibitor (Bomedemstat) (Currently in clinical trials)	(68, 69, 78, 79)
2	SCLC-N	MYC (Preclinical phase), AURKA, Oncolytic virus like Seneca valley virus inhibitors (Currently in clinical trials).	(70, 71, 72, 80)
3	SCLC-P	PARP inhibitors (Temozolomide) (Currently in Clinical trials)	(74, 81)
4	SCLC-Y	Immune check point inhibitors targeting, mTOR, PLK1, and CDK4/CDK6 pathways (early clinical stages)	(74)
5	SCLC-I	Immune check point inhibitors atezolizumab (Tecentriq) and durvalumab (Imfinzi) (Currently in Clinical trials)	(61, 82)
6	SLFN11	PARP Inhibitors (Temozolomide) (Currently in Clinical trials)	(81, 83)
7	MYC Amplification in SCLC	Aurora Kinase inhibitors AZD2811 (AURKBi) Alisertib (AURKAi) (Currently in Clinical trials)	(84, 85)

### 5.1 SCLC-A

The SCLC-A subtype is characterized by high expression of Achaete-scute homologue 1 (ASCL1), a key regulator of neuroendocrine differentiation (66, 67). From a therapeutic perspective, SCLC-A tumors may be sensitive to BCL-2 inhibitors and LSD1 inhibitors, which disrupt the interaction between LSD1 and the transcriptional repressor INSM1, thereby suppressing the expression of neuroendocrine-associated genes such as ASCL1 (62, 68, 69).

### 5.2 SCLC-N

It has an increased expression of NEUROD1, which is also critical for NE differentiation and promotes cancer cell proliferation (67). It is characterized by elevated NEUROD1 expression, often associated with MYC amplification. Potential therapeutic approaches include MYC inhibitors (70), aurora kinase A (AURKA) inhibitors (71), and oncolytic viruses like the Seneca Valley virus (72). The non-NE category consists of a group that shows negative expression of both ASCL1 and NEUROD1.

### 5.3 SCLC-P

This subtype is characterized by the expression of POU2F3 (POU Class 2 Homeobox 3), a transcription factor that is associated with chemosensory tuft cells (73). SCLC-P tumors may be responsive to poly (ADP-ribose) polymerase (PARP) inhibitors, such as veliparib (74), as well as to insulin-like growth factor 1 receptor (IGF-1R) inhibitors (45).

### 5.4 SCLC-Y

It expresses YAP1 (Yes1 Associated Transcriptional Regulator), transcriptional Coactivators involved in the Hippo signaling pathway that promotes tissue overgrowth and oncogenesis (63, 64). Therapeutic strategies may include immune checkpoint inhibitors and inhibitors targeting the mTOR, PLK1, and CDK4/CDK6 pathways (74).

### 5.5 SCLC-A2

Also referred to as NEv2, this subtype expresses HES1 (Hes Family BHLH Transcription Factor 1), a subset of ASCL1, which is frequently associated with liver metastases (75–77).

### 5.6 SCLC-I

It is a new subtype of SCLC that shows expression of immune-related genes. It is marked by high level of MHC genes, T cell attractant chemokines and immune checkpoint molecules. This subtype may be targeted by immune checkpoint inhibitors. Analysis of Impower133data shows that tumors with SCLC-I subtype respond favorably to carboplatin/etoposide/atezolizumab treatment (9, 61). SCLC-I tumors have demonstrated a favorable response to immune checkpoint inhibitors, as evidenced by retrospective analyses of clinical trials (61).

## 6 Biomarkers

The aggressive behavior of SCLC underscores the crucial need for reliable diagnosis and therapeutic strategies. Biomarkers are a diverse range of compounds derived from various sources, including blood (proteins and circulating tumor cells, circulatory nucleic acids), tissue samples, and even urine (e.g., metabolic byproducts), providing crucial information during treatment. Biomarkers act as molecular indicators of disease severity or drug response. An ideal biomarker could allow earlier diagnosis, stratify prognosis, or predict patient benefits from specific therapies (e.g., chemotherapy, immunotherapy or targeted drugs). Some biomarkers can serve as early detection tools. Mutations in RB1, a well-known tumor suppressor, are known to be associated with an increased risk of SCLC development (86). Additionally, elevated levels of the protein neuron-specific enolase (NSE) in the blood might indicate SCLC, while NSE can also be elevated in other disorders.

In addition to detection, biomarkers also play a critical role in therapy selection and treatment response prediction. CtDNA (circulating tumor DNA) or fragmented tumor DNA in the bloodstream has great potential for use in minimally invasive SCLC monitoring. Analysis of ctDNA mutations can facilitate the tracking of disease progression and therapy resistance mechanisms, potentially adjusting treatment plans for better results (87). Similarly, the presence of PD-L1 in cancer cells causes immune evasion and can predict a patient’s response to immunotherapy medications. SCLCs with high expression of PD-L1 are more likely to respond favorably to immune-boosting treatments. The landscape of SCLC biomarkers is continually evolving. The expression of Schlafen 11 (SLFN11) is being explored as a potential indicator of responsiveness to DNA-damaging agents and PARP inhibitors (88). As research continues to reveal the complexity of SCLC, these diverse indicators hold the key to gaining better knowledge of the illness and, eventually, improving patient outcomes. The following section will cover current and emerging biomarkers for SCLC, organized by their clinical role (diagnostic, prognostic, predictive) and their molecular type (genetic, epigenetic, proteomic, etc.).

### 6.1 Diagnostic biomarker

Diagnostic biomarkers provide important information regarding cancer progression and treatment effectiveness. Histopathology and Immunohistochemistry (IHC) are generally done to detect diagnostic biomarkers of SCLC, and this also helps in distinguishing it from other lung cancers. Neuroendocrine markers expressed by SCLC are synaptophysin, chromogranin A, CD56 (NCAM), and INSM1, and are usually positive for TTF-1 (thyroid transcription factor-1) (83). TTF1 is expressed in 80-90% of SCLC patients and thus helps in distinguishing from other Lung cancers (89). Various blood-based and imaging biomarkers have been investigated for non-invasive diagnosis. In SCLC, tumor cells secrete proteins such as neuron-specific enolase (NSE) and pro-gastrin-releasing peptide (ProGRP), which serve as serum tumor markers. They are known to be elevated in many SCLC patients. For instance, a recent study investigated SCLC serum positivity rates of ~61% for NSE and ~77% for ProGRP (90). Progastrin-releasing peptide (proGRP) stands out as an important therapy monitoring biomarker. Elevated proGRP levels indicate not only the existence of SCLC, but also the disease load. By analyzing proGRP levels over time, clinicians can determine the treatment response and detect possible relapses at an early stage (91).

Tumor M2‐pyruvate kinase (TuM2-PK), a new marker shown by Li et al. (2023) (90), outperformed both by showing 82.4% sensitivity and 91.1% specificity for SCLC versus 60.8%/81.1% for NSE and 77.5%/86.7% for ProGRP, respectively. The diagnostic yield was further improved when TuM2-PK was combined with NSE and ProGRP, suggesting that TuM2-PK is a promising diagnostic biomarker for SCLC (90). In practice, histological confirmation remains the gold standard to diagnose SCLC because serum markers are adjunctive. Circulating Tumor cells (CTCs), which are present in high numbers in SCLC patients, can be used as non-invasive liquid biopsy approaches (detection rates >80-90%) (93). CtDNA is also a diagnostic tool that can be used for detecting SCLC in patients, although this has not been validated yet. However, cfDNA/ctDNA (circulating fragmented DNA) analysis shows promise for non-invasive tumor detection and is being applied in trials. Tumor-derived extracellular vesicles (EVs) and Exosomes carrying RNA or SCLC-specific proteins are also being evaluated as diagnostic markers (87).

### 6.2 Prognostic biomarkers

A prognostic biomarker is a biological feature that offers insight into the tumor progression, irrespective of treatment. These biomarkers are instrumental in predicting disease progression, recurrence, or patient survival, thereby aiding clinicians in risk stratification and treatment planning (94). Prognostic biomarkers have also been identified in liquid biopsy samples. Recently, a study conducted by Mondelo-Macıá et al. (2022) (95), observed shorter progression-free (PFS) and OS strongly associated with high baseline cfDNA. According to this model, patients in higher-risk groups show significantly higher risk of early disease progression and death. Therefore, cfDNA quantity appears to be a powerful prognostic biomarker in SCLC (95).

Similarly, SLFN11, a type of DNA repair gene, correlates with chemotherapy resistance (96). Novel prognostic markers have been uncovered recently using single-cell analyses. For example, a rare subpopulation of tumor cells in SCLC with high PLCG2 expression was identified using single-cell RNA sequencing (97). The genes for metastasis and stemness were found in the subclustered “PLCG2-high” cells. Tumors in which more than 75% of cells expressed PLG2-high had significantly reduced OS. Notably, PLCG2 clusters are not limited to any subtype and hence could potentially be used as a universal prognostic marker (83).

### 6.3 Predictive biomarkers

These biological indicators assist in identifying patients who are likely to respond to a specific therapy. Predictive markers are needed urgently because treatment options beyond standard chemotherapeutic drug platinum-etoposide activity were limited. SLFN11 has the potential to act as a predictive biomarker to select SCLC patients for targeted treatments who can benefit from DNA-damage-targeting drugs, including topoisomerase and platinum-based agents and PARP inhibitors. IHC or on CTCs can be used to measure SLFN11 in patients, potentially guiding therapy choice (83, 98). TP53 and RB1 are almost universally inactivated in SCLC, and amplification of MYC-Family genes occurs in 15-20% of cases (98). SCLCs, which are MYC-driven tend to be non-NE (low ASCL1) and may respond to specific therapies. For example, preclinical studies show that Aurora kinase inhibitors could potentially target SCLC cells that have MYC gene amplification (84).

In a clinical case study, a patient with a MYCL1 fusion demonstrated a sustained response to an Aurora A inhibitor, which was followed by a favorable reaction to nivolumab as the disease progressed. NGS could be used for detecting MYC alterations and predict patients who could respond to Aurora inhibitors as well as immunotherapy response, since high MYC is linked to immune evasion phenotypes (84). DLL3 can be utilized as a biomarker for diagnosis and therapy prediction, due to its role as the target of the antibody–drug conjugate rovalpituzumab tesirine (Rova-T). Patients whose tumors were high DLL3 (≥50% cells) had a better response (38% overall response rate (ORR) in Phase I) (12). However, the clinical value of DLL3 as a predictive marker remains unproven.

### 6.4 Molecular biomarkers

They are measurable indicators of pharmacological responses to therapeutic interventions and biological processes. It consists of a range of molecules such as DNA, RNA, proteins, and metabolites that can be detected in tissues or bodily fluids to provide information for prognosis, diagnosis, and treatment selection. SCLC is genomically characterized by the loss of RB1 and TP53 (60). In approximately 20% of SCLC patients, MYC genes (MYCL, MYCN) are amplified, and their overexpression correlates with aggressive behavior and therapy sensitivity (98). In about one-third of SCLC patients, DNA repair genes (e.g. *BRCA*, *ATM*, *FANCC*) are mutated and may confer sensitivity to PARP inhibitors. Gene expression patterns at mRNA or protein level may serve as biomarkers such as high ASCL1 or NEUROD1 expression demarcates SCLC-A/N subtypes, and high POU2F3 or YAP1 would define non-NE subtypes (99). Poirier et al. (2015) (100) identified distinct DNA methylation patterns in SCLC, where EZH2 promoter methylation overexpresses EZH2, a histone methyltransferase possess strong oncogenic role and inhibiting EZH2 reduced tumor growth in SCLC models, highlighting its therapeutic potential (100). Similarly, an epigenome-wide association study on 66 cell lines of SCLC revealed SLFN11 promoter methylation correlated with resistance to DNA-damaging chemotherapy (101). CREBBP and EP300 mutations cause histone hypoacetylation, and TREX1 methylation predicts sensitivity to Aurora Kinase inhibitors, revealing molecular therapeutic targets (60).

### 6.5 MicroRNA biomarkers

**Figure 3:**
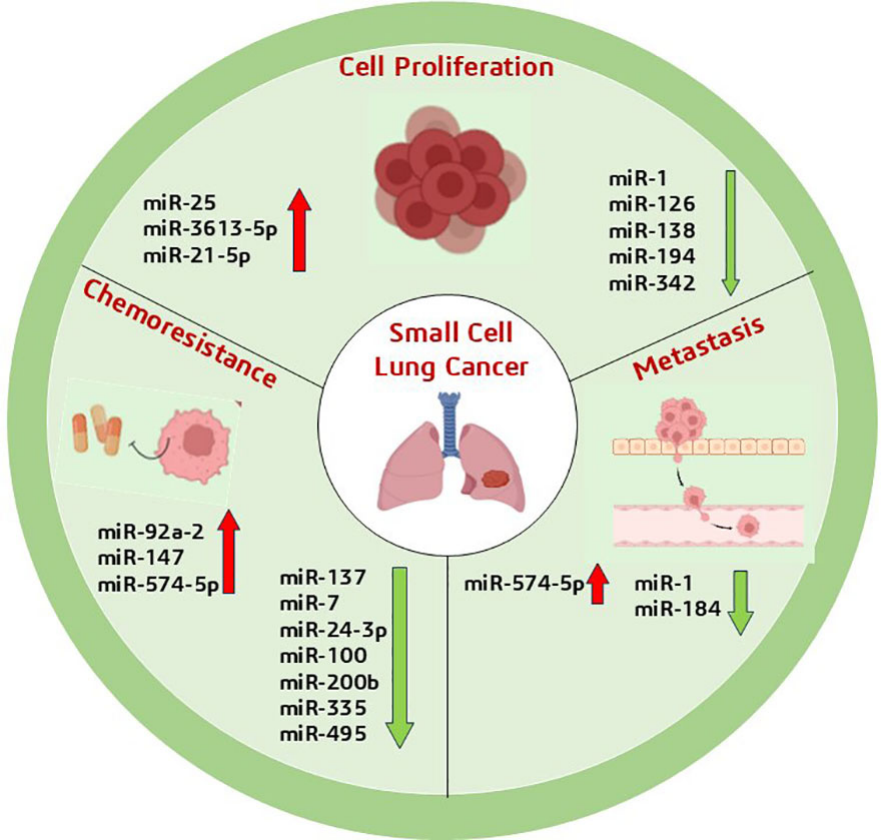
Representation of miRNAs involved in SCLC tumor progression, metastasis and chemoresistance.

MicroRNAs (miRNAs) are short non-coding RNAs that post-transcriptionally regulate gene expression. Their dysregulation has been associated with various aspects of cancer biology, including proliferation, apoptosis, metastasis, and chemoresistance in SCLC. MicroRNAs has dual oncogenic and tumor suppressive role with distinct expression in SCLC compared to NSCLC and other cancer. **Table 4** and **Figure 3** summarizes key miRNAs implicated in SCLC, some of them are miR-9, 9a, 25, 342 574-p, 126 and MiR-1 (102–111). The landscape of SCLC biomarkers is expanding. New tools like multi-omics tumor profiling, liquid biopsy, and single-cell analysis are identifying novel candidates. Many of them are in clinical or preclinical trials, but they illustrate the future directions by combining genomic, epigenomic, proteomic, and imaging data to develop new biomarker panels.

**Table 4:** MiRNA biomarkers in SCLC.

S. no.	miRNA(s)	Mechanism of action	Role as biomarker	Reference
1	miR-25	Regulates Cyclin E2, promotes cell proliferation	Oncogenic; potential diagnostic marker	(102)
2	miR-126	Targets SLC7A5, suppresses cell proliferation	Tumor suppressor; potential therapeutic marker	(103)
3	miR-138	Targets H2AX, influences DNA damage response and cell cycle	Tumor suppressor; therapeutic potential	(104)
4	miR-574-5p	Enhances metastasis via β-catenin signaling pathway	Prognostic biomarker; associated with poor outcome	(105)
5	miR-29a, miR-375	-	Diagnostic biomarker; distinguishes SCLC from NSCLC	(106)
6	miR-200b-3p, miR-3124-5p, miR-92b-5p	-	Elevated in SCLC; potential prognostic marker	(112)
7	miR-3613-5p	Promotes proliferation via AKT-MAPK pathway	Oncogenic; potential therapeutic target	(113)
8	miR-21-5p	Targets SMAD7; promotes invasion and proliferation	Oncogenic; linked to tumor aggressiveness	(107)
9	miR-608, miR-9	Upregulated in SCLC	Prognostic biomarkers	(108)
10	miR-194	Downregulated in SCLC	Tumor suppressor; associated with cancer progression	(108)
11	miR-92a, miR-147, miR-574-5p	Contribute to chemoresistance (exact mechanisms vary)	Predictive biomarker for treatment response	(109)

## 7 Cancer stem cells in SCLC

Cancer stem cells (CSCs) are subpopulation of cells within tumors that maintain quiescence, promote EMT, metastasis, therapy resistance and tumor relapse. They possess self-renewal, DNA repair mechanisms, multilineage differentiation, cell proliferation, and chemoresistance properties (114). The CSCs display high expression of key stemness-associated genes such as SOX2, MYC, and WNT1, with concurrent low expression of CD56 and CD90, thereby promoting tumor progression, chemoresistance and relapse (115). Among the markers associated with chemoresistance, tumor initiation, and stemness, CD133 is of significance; higher tumorigenicity is associated with neuropeptide receptor expression (116). In SCLC, tumor recurrence is largely attributed to CD133^+^ cells that persist after treatment and possess the ability to differentiate into diverse tumor cell populations (114). Similarly, elevated ALDH1 activity, often used in combination with CD133 to identify CSCs, enriches for CSC-like populations and enhances tumorigenicity in both NSCLC and SCLC (117).

In SCLC, CSCs exhibit high expression of SOX2, a transcription factor essential for maintaining stemness associated with poor prognosis (115). Additionally, CSCs expressing high urokinase-type plasminogen activator receptor (uPAR) show aggressive tumors with enhanced chemoresistance and tumorigenic potential (118). Epigenetics and post-transcriptional mechanisms further aid in sustaining CSC features, as evidenced by the downregulated miR-27, which preserves stemness in sphere-forming CSC-like cells (118), promoting CSC-associated DNA methylation, epigenetic regulator expression like EZH2 facilitates a stem-like, aggressive phenotype through the modulation of E2F/RB axis (119).

In CSCs, SCLC therapy resistance mechanisms are closely linked to cell surface marker expression such as CD24, CD44, and CD166. CD44, a hyaluronic acid receptor, promotes migration, invasion, and metastasis by facilitating EMT. In support, CD24 aid CSC communication through its involvement in signal transduction, migration, and adhesion (120). Similarly, CD166, the activated leukocyte adhesion molecule, supports CSCs’ migration and stemness maintenance properties. Further, EpCAM, a CSC marker, promotes migration and proliferation, mostly in epithelial-derived cancers (121). Also, resistance to SCLC therapy is associated with other CSC markers, including PODXL1, PTCH, OCT4, and BMI1. Studies on PTCH, a component of the Hedgehog signaling pathway, have shown essential for CSC self-renewal and tumor aggressiveness (120). The oncogene BMI1 stimulates self-renewal ability, thereby inhibiting senescence to increase CSC proliferation and survival (122). The transcription factor OCT4 preserves CSC pluripotency and confers resistance to therapy (123). By strengthening CSC traits, these molecular biomarkers facilitate characteristic self-renewal, proliferation, migration and drug resistance properties in SCLC (**Table 5**).

**Table 5:** CSCs-associated biomarkers in SCLC.

S. no.	CSC marker(s)	CSC features	Role as biomarker	Reference
1	SP cells (Hoechst dye exclusion), ABCG2	Self-renewal, high proliferation, drug resistance.	SP cells had lower CD56/CD90, overexpressed CSC genes (SOX2, MYC, WNT1, etc.) aids in alleviated tumor growth.	(124)
2	CD133	Chemoresistance, tumor initiation, stemness.	Increased tumorigenicity, linked to neuropeptide receptor expression.	(116)
3	ALDH1	CSC enrichment, tumorigenicity	High ALDH1 activity enriched for CSC-like populations in NSCLC and SCLC; often combined with CD133.	(117)
4	SOX2	Maintenance of stemness, poor prognosis.	Required for CSC function in SCLC; knockout/inhibition reduces CSC properties.	(115)
5	uPAR	Chemoresistance, increased tumorigenic potential.	uPAR+ cells had significantly higher tumorigenicity than uPAR− counterparts	(118)
6	miR-27 (downregulation)	Maintenance of stemness.	Sphere-forming CSC-like cells showed downregulated miR-27, sustaining stemness.	(125)
7	EZH2	Stem-like epigenetic profile, aggressive behavior.	High EZH2 expression linked to disruption of E2F/RB axis and CSC-like DNA methylation.	(119)
8	CD24	Signal transduction, migration, and adhesion.	Surface adhesion and signaling molecule implicated in CSC communication.	(120)
9	CD44	Hyaluronic acid receptor, EMT, metastasis	Linked with enhanced migration, invasion, and metastasis of CSCs.	(126)
10	CD166	Cell adhesion, migration	Activated leukocyte adhesion molecule, involved in cell migration, possibly CSC maintenance.	(120)
11	EpCAM	Proliferation, adhesion, migration.	Surface adhesion molecule, CSC marker.	(121)
12	PODXL1	Cell morphology and adhesion	Sodium-hydrogen exchanger regulatory factor; linked to stemness and aggressive behavior	(120)
13	PTCH	Developmental regulation	Part of Hedgehog pathway; disruption is associated with CSC self-renewal.	(120)
14	OCT4	Pluripotency and therapy resistance	POU domain transcription factor is essential for maintaining CSC pluripotency.	(123)
15	BMI1	Self-renewal and senescence suppression	Oncogene promoting CSC proliferation and survival.	(122)

### 7.1 CSCs and immune response in SCLC

The SCLC tumors exhibit strong immune evasion mainly due to reduced MHC-I expression, which impairs T-cell recognition and antigen presentation. Neuroendocrine subtypes such as SCLC-A (ASCL1+) and SCLC-N (NEUROD1+) have shown strong MHC-I repression, making PD-L1 blockade largely ineffective (127). This limits the use of immune checkpoint inhibitors (ICIs) in SCLC therapeutics. The aggressiveness and highly metastatic nature of SCLC are linked to CSC-like behavior and an immunosuppressive tumor microenvironment (TME) that makes immunotherapy ineffective in combating the disease (114). Various immune regulatory molecules like cytokines (IL-6, IL-8, TNF-α, INF-γ) play a significant role in the induction of stemness in SCLC due to their presence in the TME. Studies on H446 (SCLC cell line), sphere-forming CSCs, showed high self-renewal, migratory, and tumorigenicity potential with increased IL-8 expression. Inhibiting IL-8 expression reduces stemness and tumor growth, while IL-8 expression rescue reverses the aggressive phenotype, highlighting its potential as a therapeutic target (128). Tumor-associated macrophages (TAMs) and Myeloid-derived suppressor cells (MDSCs) suppress anti-tumor immunity via releasing cytokines and growth factors such as IL-10 and TGF-β, thereby inhibiting cytotoxic CD8^+^ T-cells and natural killer (NK) cells to sustain CSCs promoting tumor growth and inhibiting immune checkpoints (129).

## 8 Molecular biomarkers driving precision oncology

Biomarkers are pivotal to modern precision therapy in lung cancer, influencing diagnosis, prognosis, and therapy selections. In NSCLC, it is driven by specific, targetable oncogenic mutations that underpin precision oncology. In contrast, personalization in SCLC is evolving with biomarker-guided therapy models that do not rely on a single dominant driver mutation; instead, it is based on molecular subtypes, tumor immune microenvironment (TME), DNA damage response (DDR), and liquid biopsy-based monitoring (130).

Four canonical Transcription factor (TF)-based subtypes (ASCL1, NEUROD1, POU2F3, YAP1) and an inflamed SCLC-I/Y(I) immune-rich subtype form the backbone of emerging precision strategies in SCLC (131). Multi-omics and mutational-signature clustering further revealed the cause of ineffective therapeutics, mainly due to heterogeneity in SCLC tumors characterized by vulnerabilities to DDR-targeting agents, aurora-kinase inhibitors, and immune checkpoint blockade. Emerging studies suggest that MYC-high SCLC tumors exhibit selective sensitivity to aurora kinase A/B (AURKA/AURKB) inhibitors and the SCLC-I subtype is vulnerable to immune checkpoint inhibitors (ICIs) (132). Traditional immunotherapy biomarkers such as PD-L1 and Tumor Mutational Burden (TMB), have shown inconsistent predictive value for ICI response in SCLC and ES-SCLC. Composite markers integrating TF subtype, TME signatures and chemokine indices show more promise for ICI enrichment (133).

DLL3 has emerged as a cell surface biomarker in SCLC, with higher expression in tumors compared to normal tissues. This has enabled multiple DLL3-directed treatment approaches, including antibody–drug conjugates (such as Rovalpituzumab tesirine (Rova-T)), bispecific T-cell engagers (Tarlatamab-dlle), and DLL3-targeted CAR-T/NK cells. While early Rova-T data suggested better outcomes in DLL3-high tumors, supporting its use as a stratification marker, later trials showed a weaker association and responses to Tarlatamab-dlle regardless of DLL3 expression levels (134, 135).

Liquid biopsy provides a solid platform for real-time precision in SCLC. Circulating tumor DNA (ctDNA) and circulating tumor cells (CTCs) are evidently detected in most cancer patients and can be frequently monitored using a minimally invasive approach. This enables dynamic tracking of tumor burden, treatment response and resistance. Liquid biopsy facilitates longitudinal, biomarker-guided adaptation of therapy response when repeat tissue biopsies are not achievable. In SCLC, longitudinal ctDNA analyses underscore ctDNA as a marker for therapy response and progression. While CTCs in SCLC can be molecularly characterized for therapeutic markers like DLL3 and SLFN11, allowing the non-invasive evaluation of eligibility for DLL3-targeted therapies and offering a platform for monitoring these biomarkers over the treatment timeline (136, 137). Thus, precision oncology in SCLC integrates all these approaches to guide personalized treatment strategies. Ultimately, this framework facilitates patient-centered care and improves disease outcomes.

### 8.1 Clinical advances in biomarkers for targeted therapeutics

PDL-1, a predictive biomarker, is currently explored extensively for immunotherapy, specifically in relevance to immune checkpoint inhibitor-based clinical trials. Recent clinical trials in limited-stage small-cell lung cancer (LS-SCLC) on evaluating the PDL-1 inhibitors, such as Durvalumab and Atezolizumab, have shown an increase in survival outcomes, in combination with chemotherapy and concurrent chemoradiotherapy (CCRT). In parallel, an ongoing clinical study is directed towards systematic assessment of PDL-1-guided immunotherapy to refine personalized treatment options to improve the overall clinical outcome of LS-SCLC patients (137).

Schlafen 11 (SLFN11), a notable biomarker in SCLC, has been shown to increase tumor sensitivity to PARP inhibitors and to DNA-damaging treatments, including platinum-based chemotherapeutic drugs. SLFN11-positive patients have shown improved clinical outcomes and longer progression-free survival in a phase-II clinical trial evaluating the combination of temozolomide and Veliparib (138). Also, a recent clinical study in SLFN11-positive patients has shown improved progression-free survival with atezolizumab plus talazoparib, underscoring its profound importance in SCLC therapeutics (139).

DLL3-clinically targeted therapies in SCLC are rapidly increasing, reflecting a biomarker-driven treatment prototype with diverse remedial platforms that are being actively examined across different stages of clinical development. DLL3 has evolved as a cancer-specific antigen, paving the way for the development of targeted therapeutic strategies such as antibody-drug conjugates, including Rovalpituzumab tesirine. Targeting DLL3 with bispecific T-Cell engagers like tarlatamab has shown promising clinical efficacy and has also escalated into late-phase trials for evaluating the overall survival rate. Also, advancing next-generation targeted approaches, including Tri-specific T-cell engagers and therapies such as CAR-NK and CAR-T-cell therapy, are being investigated in early-phase clinical studies to boost antitumor immune responses. These clinical interventions typically evaluate a wide spectrum of endpoints, including safety, overall survival, dose optimization and response rates, highlighting the development of DDL3-targeted therapies from preclinical to clinically effective interventions (140). Furthermore, **Table 6** provides a comprehensive overview of current clinical studies on biomarker-guided therapeutics, outlining their potential benefits along with associated limitations.

**Table 6:** Current clinical landscape of biomarker-guided therapeutics in SCLC.

S. no.	Therapeutic strategy	Targets	Therapeutic agents	Benefits	Limitations	Clinical trial ID		Reference
						Ongoing	Completed	
1.	Anti-Angiogenic Agent	VEGF, VEGFR, FGFR, PDGFR, c-Kit, VEGFR2, c-SRC tyrosine kinases.	Bevacizumab	It shows improved progression-free survival (PFS) in combination with chemotherapy	Bevacizumab does not improve the overall clinical outcomes.	–	NCT00403403, NCT04730999	(141–144)
			Anlotinib	Improved PFS in ES-SCLC.	Lacks large, randomized control groups.	NCT04165330-Phase-Ib	NCT04660097, NCT03059797 NCT04055792	
			Cediranib	–	–	NCT02498613-Phase-II	–	
2.	DNA Damage response inhibitors (DDR).	PARP	Olaparib	Moderate Anti-tumor activity.	Less improvement in PFS.	NCT04728230-Phase I/II, NCT03923270-Phase-I.	NCT03532880, NCT02769962, NCT04939662	(92, 142, 144–147)
			Talazoparib	Modest anti-tumor activity.	Poor durability.	NCT04334941-Phase II, NCT03672773-Phase II	NCT01286987	
			Rucaparib	Combined therapy shows promising activity.	Low sample Size.	–	NCT03958045	
			Fluzoparib	DNA damage repair inhibition.	Limited study data.	–	NCT04400188	
			Veliparib	Improved response rate and biomarker-based treatment.	No significant improvement in PFS and overall survival.	–	NCT01638546, NCT01642251	
3.	Chimeric antigen receptor (CAR)-T-cell therapy	DLL3/CD3	Tarlatamab (AMG757)	–	–	NCT03319940-Phase-I, NCT03319940-Phase-II, NCT05060016-Phase-II	–	(142, 144, 148, 149)
			HPN328	–	–	NCT04471727- Phase-I/II	–	
			BI 764532	–	–	NCT04429087	–	
			AMG119, 757	CAR-T retention in relapsed SCLC.	Median efficacy with a small sample size.	–	NCT03392064, NCT04885998	
			ROVA-T	Targeted anti-tumor activity against DLL3.	Significant Toxicity.	–	NCT03061812, NCT02674568	
4.	Epigenetic regulation	EZH2 and LSD1	EZH2 and LSD1 inhibitors (DS-3201b)	–	–	–	–	(142, 144)
			Tazenetostat plus topotecan and pembrolizumab	–	–	NCT05353439-Phase-I	–	
5.	Antibody-Drug Conjugates	TROP-2	Sacituzumab govitecan (IMMU-132)	Promising responses were observed even in heavily pre-treated SCLC patients.	Significant toxicities like neutropenia, diarrhea and fatigue.	NCT04826341-Phase I/II	NCT01631552	(142, 144, 150)
6.	Cell surface receptors	Fucosyl-Monosialoganglioside 1 (Fuc-GM1)	BMS-986012	Selective tumor targeting in SCLC due to the presence of Fuc-GM1.	Combined therapy shows more efficacy than monotherapy.	NCT04702880-Phase-II	–	(151)

#### 8.1.1 Anti-angiogenic agents

Bevacizumab: By targeting vascular endothelial growth factor (VEGF), bevacizumab, a humanized monoclonal antibody, inhibits the formation of new blood vessels (152). Platinum-based chemotherapy and chemoradiation have been tested in conjunction with bevacizumab (VEGF inhibitor). Although preliminary research indicated improvements in PFS, risks like the development of tracheoesophageal fistula were noted, and there was no discernible improvement in OS (153–156). Limited advantages were also demonstrated by aflibercept (VEGF-A and VEGF-B inhibitor) and sunitinib (VEGF TKI). In clinical trials, aflibercept did not improve OS, but sunitinib showed a slight improvement in PFS (157, 158).

#### 8.1.2 DNA-damaging agents/DNA damage response inhibitors

SCLC generally has a high tumor mutational burden (TMB), but immunotherapy response has been limited, and the major cause behind this is defective antigen presentation and reduced T cell infiltration, and significant immunosuppression in the TME (159). Inhibitors of PARP, including talazoparib, olaparib, and veliparib, have been promising, particularly in conjunction with temozolomide (TMZ). In relapsed SCLC, combination treatments have shown promising ORR of about ~40% (137, 144, 160, 161). Increased sensitivity towards PARP inhibitors is correlated with SLFN11, a crucial biomarker. Clinical trials such as SWOG 1929 showed that patients with SLFN11-positive tumors benefited more from PARP inhibitor therapies (137, 144, 162). Although definitive clinical data are still pending, ATR and CHK1 inhibitors are being studied for SCLC patients, especially in tumors lacking in TP53 or ATM (137, 163).

#### 8.1.3 Epigenetic therapeutics

Epigenetic dysregulation is a hallmark of SCLC, offering new therapeutic targets. Inhibitors of EZH2: Often in SCLC, histone methyltransferase EZH2 is upregulated, therefore causing transcriptional silencing of tumor suppressor genes. Preclinical research has shown that EZH2 inhibition can inhibit the proliferation of SCLC cells and reactivate tumor suppressor genes (137, 164). PF-06821497 is one such inhibitor that is in Phase 1 clinical trials and is being tested for treating SCLC along with chemotherapeutic drug irinotecan (137, 165).

Lysine demethylase 1 (LSD1, also known as KDM1A), a histone-modifying enzyme, has been proposed as a potential therapeutic target in SCLC. When LSD1 is inhibited, NOTCH is activated, ASCL1 is suppressed, and MHC1 expression rises (137, 62). LSD1 blockade increased the susceptibility of SCLC tumors to immunotherapy when paired with PD-1 inhibitors (166). In primary samples and SCLC cell lines, the LSD1-targeted medication GSK2879552 has shown potent antitumor activity. Due to significant toxicity and insufficient efficacy, the study of GSK2879552 in relapsed/refractory SCLC was terminated (167).

#### 8.1.4 Antibody drug conjugates

The most recent development in precision oncology is represented by antibody–drug conjugates, which target specific cell surface receptors or proteins in many cancers, including SCLC. ADCs consist of three parts (1): a cytotoxic drug payload, (2) a monoclonal antibody that will bind to an antigen on the surface of the tumor cell selectively, and (3) a linker that joins the two (168). Thus, multifunctional ADCs can target tumor cells precisely, internalize, and fuse with lysosomes to release a cytotoxic agent that kills the cells and reduces off-target effects.

Numerous epithelial cancers express the trophoblast cell surface antigen (TROP2), and overexpression of this protein is associated with a lower chance of survival. TROP 2 has been found to be highly expressed in 18% of high-grade NE tumors, including 10% of SCLC (169). Sacituzumab govitecan (IMMU-132), a first-in-class anti-TROP2 antibody–drug conjugate (ADC), consists of an anti-TROP2 monoclonal antibody linked to the topoisomerase I inhibitor SN-38. In a cohort of 62 pre-treated SCLC patients, this ADC demonstrated an ORR of 18%, a median duration of response (DOR) of 5.7 months, PFS of 3.7 months, and OS of 7.1 months (150). Another candidate target for SCLC is SEZ6, a cell surface protein predominantly expressed in neuroendocrine tumors and largely absent in normal tissues (170). Ifinatamab deruxtecan (I-DXd) is a novel antibody–drug conjugate (ADC) composed of an anti-B7-H3 antibody linked to Dxd, a DNA topoisomerase inhibitor. In patients with various heavily pretreated cancers, including esophageal, prostate, and lung cancers, I-DXd has shown promising durable responses. Among 19 SCLC patients, 53% achieved confirmed responses, with a median DOR of 5.5 months (171).

#### 8.1.5 Other potential treatments

CD47, a cell surface ligand overexpressed in several cancers, plays a key role in immune evasion by sending a “don’t eat me” signal to macrophages, thereby preventing phagocytosis. Given the frequent use of radiation therapy alongside systemic treatments for both palliative and curative purposes in SCLC, preclinical studies have examined the interaction between radiation and anti-CD47 therapy in SCLC mouse models. These studies demonstrated that anti-CD47 blockade not only enhanced the anti-tumor effect of radiation but also reduced tumor growth outside the radiation field and conferred resistance to re-challenge with freshly injected SCLC cells (172).

Researchers discovered that SCLC is responsive to transcription-targeting medications, particularly CDK7 inhibitors, which is a promising new approach for the treatment of SCLC (173). One such drug SY-5609, an oral CDK7 inhibitor has been approved by FDA (174). The monosialoganglioside fucosyl-GM1 (FucGM1) is minimally expressed in normal tissues but highly expressed on the surface of SCLC cells. BMS-986012, a first-in-class, fully human, nonfucosylated IgG1 monoclonal antibody, targets FucGM1. In a phase 1 study, BMS-986012 demonstrated limited monotherapy activity (ORR 4%, n = 77) but showed notable efficacy in SCLC mouse xenograft models. When combined with the anti-PD-1 antibody nivolumab, the ORR increased to 38% (n = 29). Currently, a study is ongoing evaluating BMS-986012 in combination with nivolumab and chemotherapy as first-line treatment for extensive-stage SCLC (175).

## 9 Research constraints and implications

Despite recent progress, several limitations continue to affect the clinical management of small-cell lung cancer (SCLC). The high degree of tumor heterogeneity remains a major challenge, limiting the identification of reliable biomarkers and effective targeted therapies. Although multiple biomarkers have been proposed, only a few have been validated for routine clinical use (83). In addition, many therapeutic strategies that show promise in preclinical studies have demonstrated limited or variable success in clinical settings, reflecting the complexity of the disease (12, 62). Resistance to chemotherapy and immunotherapy further contributes to poor patient outcomes.

While molecular subtyping has improved our understanding of SCLC, its translation into clinical practice is still evolving. Future research should therefore focus on integrating multi-omics approaches, improving translational models, and conducting well-designed clinical trials to validate emerging biomarkers and therapeutic targets. Addressing these limitations will be essential for advancing personalized treatment strategies and improving outcomes in SCLC patients.

## 10 Conclusion

This comprehensive review on complete investigation of small cell lung cancer (SCLC) offers light on its complicated character, which is influenced by multiple genetic changes, tumoral heterogeneity, and tumor development. Understanding SCLC’s intricate processes, such as genetic mutations, various subtypes defined by important regulators, and the participation of cancer stem cells (CSCs), is critical for developing effective diagnostic and treatment approaches. The discovery of CSCs inside SCLC tumors, as well as their connection with treatment resistance, emphasizes the need to focus on these cells to enhance patient OS outcomes. Biomarkers, both intracellular and extracellular, provide important information for early identification, tumor characterization, and disease progression and therapy response monitoring. Furthermore, the identification of SCLC subtypes based on the predominant expression of critical regulators such as ASCL1, NEUROD1, YAP1, POU2F3, and ATOH1 shows the heterogeneity of this malignancy and offers personalized treatment approaches based on subtype-specific vulnerabilities. This increased understanding of SCLC’s molecular landscape and subtype-specific traits lays the door for the development of tailored medicines and precision medicine initiatives to improve patient outcomes. In conclusion, the multimodal approach to investigating SCLC, which includes genetic, molecular, cellular, and clinical elements, provides a complete framework for understanding its intricacies and guiding future research and therapeutic approaches. By exploiting this information and utilizing emerging technology and therapeutic modalities, we may work towards more effective therapies, improved prognostic tools, and, eventually, better outcomes for patients facing this aggressive type of lung cancer. The paper highlights how precision oncology is reshaping small cell lung cancer (SCLC) treatment by pairing targeted therapies with specific biomarkers. Key examples include PARP inhibitors combined with SLFN11 positivity, Aurora kinase inhibitors in MYC-amplified tumors, DLL3-directed therapies (such as CAR-T and BiTEs) in DLL3-overexpressing cancers, and epigenetic drugs like EZH2 or LSD1 inhibitors in tumors with promoter methylation or ASCL1-driven neuroendocrine programs. Antibody–drug conjugates against TROP2, SEZ6, and B7-H3, as well as CD47 blockade with radiation, are also emerging strategies. In transformed SCLC, where EGFR mutations are retained but TP53/RB1 are lost, and neuroendocrine markers are acquired, platinum–etoposide remains the backbone, but combinations with EGFR TKIs or epigenetic modulators may extend benefit. Overall, the paper emphasizes biomarker-guided therapy as the path forward for both *de novo* and transformed SCLC, aiming to overcome resistance and lineage plasticity.

## Glossary

SCLC: Small cell lung cancer
NSCLC: Non small cell lung cancer
OS: Overall survival
FDA: Food and Drug Administration
PFS: Progression free survival
IHC: Immunohistochemistry
ctDNA: Circulating tumor DNA
CD: Cluster of Differentiation
CTCs: Circulating tumor cells
cfDNA: Circulating fragmented DNA
EVs: Extracellular Vesicles
NGS: Next generation sequencing
ORR: Overall response rate
WHO: World Health Organization
CSC: Cancer Stem Cell
DDR: DNA Damage Response
ADC: Antibody drug conjugate
CAR: Chimeric antigen receptor
MHC: Major Histocompatibility complex
miRNAs: MicroRNAs
